# The perceptions and experiences of women who achieved and did not achieve a waterbirth

**DOI:** 10.1186/s12884-017-1637-5

**Published:** 2018-01-10

**Authors:** Lucy Lewis, Yvonne L. Hauck, Caroline Crichton, Courtney Barnes, Corrinne Poletti, Helen Overing, Louise Keyes, Brooke Thomson

**Affiliations:** 1 School of Nursing, Midwifery and Paramedicine, Curtin University, Bentley, Perth, Western Australia 6102 Australia; 2 Department of Nursing and Midwifery Education and Research, King Edward Memorial Hospital, Subiaco, Western Australia Australia; 3 King Edward Memorial Hospital, Subiaco, Western Australia Australia

**Keywords:** Waterbirth, Midwifery-led care, Intrapartum care, Birth experience

## Abstract

**Background:**

There is a gap in knowledge and understanding relating to the experiences of women exposed to the opportunity of waterbirth. Our aim was to explore the perceptions and experiences of women who achieved or did not achieve their planned waterbirth.

**Methods:**

An exploratory design using critical incident techniques was conducted between December 2015 and July 2016, in the birth centre of the tertiary public maternity hospital in Western Australia. Women were telephoned 6 weeks post birth. Demographic data included: age; education; parity; and previous birth mode. Women were also asked the following: what made you choose to plan a waterbirth?; what do you think contributed to you having (or not having) a waterbirth?; and which three words would you use to describe your birth experience? Frequency distributions and univariate comparisons were employed for quantitative data. Thematic analysis was undertaken to extract common themes from the interviews.

**Results:**

A total of 31% (93 of 296) of women achieved a waterbirth and 69% (203 of 296) did not. Multiparous women were more likely to achieve a waterbirth (57% vs 32%; *p* < 0.001). Women who achieved a waterbirth were less likely to have planned a waterbirth for pain relief (38% vs 52%; *p* = 0.24). The primary reasons women gave for planning a waterbirth were: pain relief; they liked the idea; it was associated with a natural birth; it provided a relaxing environment; and it was recommended. Two fifths (40%) of women who achieved a waterbirth suggested support was the primary reason they achieved their waterbirth, with the midwife named as the primary support person by 34 of 37 women. Most (66%) women who did not achieve a waterbirth perceived this was because they experienced an obstetric complication. The words women used to describe their birth were coded as: affirming; distressing; enduring; natural; quick; empowering; and long.

**Conclusions:**

Immersion in water for birth facilitates a shift of focus from high risk obstetric-led care to low risk midwifery-led care. It also facilitates evidence based, respectful midwifery care which in turn optimises the potential for women to view their birthing experience through a positive lens.

## Background

Despite the cumulative body of evidence around the safety and efficacy of maternal and neonatal outcomes associated with waterbirth, there is a gap in knowledge and understanding relating to the experiences of women who birth in water. No studies were identified comparing the perceptions and experiences of women who achieved or did not achieve their planned waterbirth. Additionally, a meta-analysis of 12 randomised controlled trials (RCTs) including 3243 women [1] found limited, inconclusive evidence in relation to this topic. A literature search including 38 studies of 31,000 women [2] suggested waterbirth was associated with high levels of maternal satisfaction with pain relief and childbirth experience.

Labour and birth is an individualised experience incorporating physiological, emotional and psychosocial factors. As the trajectory of labour can be unpredictable [1], intrapartum care within a hospital setting often focuses on the risk status of the woman rather than viewing birth as a normal physiological process. Labouring and birthing in water facilitates a shift from high risk obstetric-led care to low risk midwifery-led care, where care is provided by an individual midwife or team of midwives [3, 4]. Midwifery-led models of care are based on the philosophy that pregnancy and birth are normal life events [3] as the majority of women and their babies remain healthy, with no comorbidities or risk factors [5].

Although models of midwifery-led continuity of care can involve a team of midwives, maternity care is usually provided within a caseload model [4]. Generally, a prerequisite for acceptance into caseload midwifery is that women are obstetrically and medically low risk [3, 4, 6]. Midwives providing care to women within this model often work alongside another midwife or small team of midwives who are known to the woman and can provide backup if the primary midwife is not available [4, 6]. This means midwives can provide continuity of care 24 h a day across hospital settings, free standing birth centres and women’s homes [4, 7]. Generally each midwife has a caseload of between 32 and 40 women per annum [6, 7].

A meta-analysis of midwife-led continuity models versus other models of maternity care (including 15 RCTs with 17,674 women) found women rated midwifery-led continuity of care models highly in terms of their satisfaction [3]. This finding is not surprising as women who receive midwifery-led maternity care are likely to have a midwife they know for their birth, a spontaneous vaginal birth and experience less intervention [3]. Indeed, *The Lancet’s* Midwifery series highlights improved outcomes for women and their infants when care is provided by midwives who are educated [8, 9], regulated [8, 9] and provide respectful evidence based care [10]. This care model empowers women to realise their potential and view labour through a positive lens [1, 5, 11].

Empowerment is a key component of woman centred care and the impetus to normalise birth [1]. Women who meet the criteria to labour and birth in water often share the philosophy that pregnancy and birth are normal healthy life events [1]. This salutogenic approach to birth embraces positive health and wellbeing rather than negative pathogenic outcomes [5, 11]. It has been asserted that a consequence of this philosophy is the promotion of increased satisfaction [12–15], as several studies exploring satisfaction of women with uncomplicated pregnancies confirmed high satisfaction rates when women remained in their primary care setting rather than be transferred to a secondary or tertiary care setting [12–15].

Other work by members of this research team explored women’s perceptions of the first ‘no exit’ Midwifery Group Practice in a West Australian (WA) tertiary obstetric hospital; where the midwife continued to accompany the woman at every episode of care. The service was found to be highly acceptable to women with 98% of the 232 women surveyed recommending the service. The findings also reinforced the value of continuity of care within maternity services [6]. Additional WA research at a birth centre explored the transfer journey from the birth centre to the tertiary maternity hospital from the view of the woman, her partner and the midwife [16]. Findings revealed that experiences of intrapartum transfer were unique to each member of the triad and were not always positive. Adjusting to and accepting the medical model of care after transfer facilitated a positive experience [16].

The practice of immersion in water for labour and birth is predominantly under the domain of midwives working with low risk women within midwifery models of care. Currently, there is a gap in our knowledge and understanding of the experiences of women exposed to the opportunity of water immersion for labour and birth. No studies were identified which compared the perceptions and experiences of women who achieved or did not achieve their planned waterbirth. Therefore, our aim was to explore the perceptions and experiences of these women.

## Method

An exploratory design using critical incident techniques was utilised. This technique allows experiences of direct behaviour that have critical significance and meet methodically defined criteria to be evaluated [17]. The technique has been previously utilised to evaluate consumer expectations and perceptions in health care [18, 19]. The incident has to be clearly defined, as it is the basic unit of analysis [17]. For this study the unit was women’s experiences around their planned waterbirth. Data were collected from the participant’s perspective and in their own words. Generally, 100 critical incidents are recommended [20]. However, as this methodology is qualitative the final sample size is determined by data saturation. Ethics approval was gained from the Women and Newborn Ethics Committee (Approval Number 2016123QK) at the study centre.

### Participants and data collection

The study was conducted between December 2015 and July 2016 at the sole tertiary public maternity hospital in WA, which has approximately 5200 births annually. There has been an exponential rise in the number of women embracing waterbirth in WA. Perinatal data collected by the Obstetrics and Gynaecology Clinical Care Unit at King Edward Memorial Hospital (the study centre) found 73 women achieved a waterbirth in 2010, whilst in 2016, 4% (228 of 5189) of all infants and 5% (228 of 4402) of infants ≥37 weeks gestation were born underwater.

At the time of the study, women wanting to labour or birth in water were cared for by midwives working within a low-risk midwifery-led caseload model of care [4], based in a birth centre; a building adjacent to the tertiary maternity hospital. All women who planned to birth in water and had signed an ‘Agreement for use of water for birth’ were included. An information sheet outlining the study and informing women they may be contacted post birth was given to women at 37 weeks gestation by their midwife. Six weeks post birth women were telephoned by a research midwife not involved in their clinical care and invited to participate in the research. The research team then made two further attempts 1 week apart, to contact women. The study purpose was explained to all women and verbal agreement to participate in a telephone interview with the research team was considered ‘implied consent’.

Prior to commencing the interview questions, women were asked about their: age; education level; parity; and previous birth mode. Women were also asked if they had achieved their planned waterbirth. Verbatim responses were then gleaned in relation to the following open ended questions: what made you choose to plan a waterbirth? and what do you think contributed to you having (or not having) a waterbirth? In addition, women were asked to identify three words to describe their birth experience. As the interviews were not audio recorded, once the woman shared her perceptions, the interviewer provided a verbal summary of her responses which were read back to her after each question, giving the opportunity for reflection and enabling the woman to add anything she may have missed. Finally, women were then asked to rank the top three responses they perceived were most important.

### Data analysis

#### Quantitative data: demographic characteristics and frequency of women’s responses

Descriptive statistics were based on medians, interquartile ranges and ranges for continuous data (such as maternal age) and frequency distributions for categorical data (such as parity). Univariate comparisons between the groups (those women who achieved a waterbirth and those who did not) were performed using Chi-square tests. *P*-values <0.05 were considered statistically significant. SPSS statistical software (version 21, IBM SPSS Statistics for Windows, Version 20.0 Armonk, NY: IBM Corp) was used for data analysis.

#### Qualitative analysis: open ended questions and birth descriptors

A systematic process of thematic analysis was used to determine what women perceived as important for their planned waterbirth [21]. From the verbatim responses of their birth experiences in relation to the two questions (what made you choose to plan a waterbirth? and what do you think contributed to you having or not having a waterbirth?) and the words women used to describe their birth experience, the research team extracted common themes, patterns and similarities around the women’s perceptions. Four members of the research team independently grouped the data into common themes the women had identified as being instrumental in assisting their decision to birth in water and the factors they perceived had contributed to achieving (or not achieving) their waterbirth. The researchers shared their preliminary analysis based upon the women’s responses and negotiation then occurred to determine final themes that reflected their responses [22]. Once an agreement was reached with the final themes, they were used to determine the citation frequency from women’s ranking of their importance. The responses were entered into an Excel database and recoded to match the final themes agreed upon by the research team. Descriptive statistics comprising of frequency distributions were then calculated. Findings are supported with verbatim quotes from the women. A coding system (P1 to P296) was used for each woman to ensure their confidentiality and privacy. Additionally, each quote was allocated a postfix to indicate their parity (‘P’ for being primiparous and ‘M’ for being multiparous) and if they achieved a waterbirth (‘Y’ for did achieve a waterbirth and ‘N’ for did not achieve a waterbirth).

## Results

### Quantitative results

Between December 2015 and July 2016, 342 women were identified who had signed an ‘Agreement for use of water for birth’; 86% (296 of 342) agreed to participate in the study. A total of 31% (93 of 296) of women achieved a waterbirth and 69% (203 of 296) did not. The mean age of women was 31 years. Multiparous women were more likely to achieve a waterbirth (57% vs 32%; *p* < 0.001). Women who achieved a waterbirth were less likely to have planned a waterbirth for pain relief (38% vs 52%; *p* = 0.24). Most (69%) women had an undergraduate or postgraduate degree (Table 1).

**Table 1:** Characteristics of women who achieved or did not achieve a waterbirth

Characteristic	Birthed in water	Did not birth in water	P Value	Total
	n = 93 (31%)	n = 203 = (69%)		n = 296
	n (%)	n (%)		n (%)
Maternal age	31/31 (28-33) [20-47]	31/31 (29-33) [19-43]	0.065	31/31 (29-33) [19-37]
Primiparous	40 (43)	139 (68)	<0.001	179 (61)
Multiparous	53 (57)	64 (32)		117 (39)
Multiparous first birth^a^				
Spontaneous vaginal	45 (85)	50 (79)	0.440	95 (82)
Assisted vaginal/caesarean birth	8 (15)	13 (21)		21 (18)
Education				
High school/Diploma	31 (33)	60 (30)	0.513	91 (31)
Undergraduate/postgraduate education	62 (67)	143 (70)		205 (69)
Primary reason chose waterbirth				
Pain relief	35 (38)	105 (52)	0.024	140 (47)

^a^Included n = 18 women who had a previous waterbirth

### Qualitative results

#### What made women choose to plan a waterbirth

**Fig. 1:**
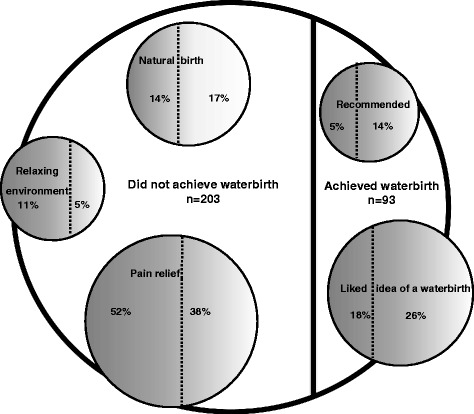
What made you plan a waterbirth

Figure 1 highlights what influenced women to plan a waterbirth. Ranked words were analysed and presented as a bubble graph where the large background circle represents the entire population of women surveyed. A thick vertical solid line shows the divide between women who achieved a waterbirth and those who did not. Within the large background circle smaller circles represent the primary sentiment expressed by women in response to what contributed to planning a waterbirth. A broken vertical line shows the divide between those women who achieved a waterbirth (on the right) and those who did not (on the left). In addition, the frequency of the response cited is reflected by the size of the circle with more frequent responses represented by larger circles. The primary reasons women gave for planning a waterbirth were: pain relief; they liked the idea; it was associated with a natural birth; it provided a relaxing environment; and it was recommended.

#### Pain relief

Just under half (47%; 139 of 296) of all women surveyed, 38% (35 of 93) of women who did and 52% (105 of 203) of women who did not birth in water, chose a waterbirth as they perceived it would provide them with pain relief in labour. Many women thought water immersion would help them avoid an epidural: ‘*I heard it [waterbirth] provided 75% pain relief, don't like taking medication, didn't want epidural*’ (P47^N,M^), another ‘*knew I didn't want epidural, fearful of epidural*’ (P249^N,P^) and ‘*I didn't want epidural or drugs, heard water was the next best thing*’ (P187^N,P^). There was a perception that water immersion would provide ‘*natural pain management*’ (P237^Y,M^) and ‘*prevent pharmaceutical drug use with water as an alternative*’ (P281^Y,P^) through ‘*pain relief from freedom to move’* (P236^Y,M^). One woman just ‘*really wanted to avoid ‘hardcore’ pain relief,’ I thought it [waterbirth] might be a form of relaxation’* (P213^N,M^). Another was content to use water immersion in labour but *‘not ‘hellbent’ on having a waterbirth, I just thought it might be a useful tool as had heard it was even better than some pharmacological pain relief’* (P204 ^N,P^). Some women also thought water would help them deal with uterine contractions as ‘*I get bad menstrual pain- bath helps so felt would be good pain relief in labour* (P137 ^N,P,)^ and ‘*I use water for pain relief in everyday life, e.g. my period*’ (P62^Y,P^).

#### Liked the idea

A total of 21% (44 of 296) of all women surveyed, 26% (24 of 93) of women who did and18% (36 of 203) of women who did not birth in water, chose a waterbirth because they liked the idea. Some women were prompted to opt for a waterbirth because they had ‘*seen it [waterbirth] online*’ (P257 ^N,P^)’ or ‘*saw videos and wanted a beautiful experience*’ (P290 ^N,P^) and ‘*saw on One Born Every Minute* [the television show]*, looks wonderful*’ (P189 ^Y,M^). There were a couple of women who had ‘*always wanted a waterbirth*’ (P70^Y,P^) and ‘*I have always wanted a waterbirth, a waterbirth seemed more natural to me*’ (P208 ^Y,M^). One woman described how she liked the idea of a waterbirth as she perceived ‘*it’s a similar environment to the womb and calm for baby*’ (P181 ^N,P^) while another explained ‘*I wanted to be able to deliver my own baby, I love the idea of being immersed in water and baby being delivered into water*’ (P94 ^N,P^). Some women had previously experienced a waterbirth and wanted to repeat the experience. ‘*I have birthed all my babies in water… I feel that waterbirth allows for a natural birth process*’ (P209 ^Y,M^) and ‘*my first baby was a beautiful experience I wanted to repeat. Scooping baby up onto chest from water such a great moment*’ (P230 ^Y,M^). One woman explained how she had ‘*laboured in water for past births, I liked the reduced gravity, but got out to birth*’ (P256 ^N,M^). Finally another stated ‘*This was the last baby I planned and so wanted it to be memorable, I wanted a different experience than last time*’ (P65^N,M^).

#### Natural birth

A total of 15% (44 of 296) of all women surveyed, 17% (16 of 93) of women who did and 14% (28 of 203) of women who did not birth in water, chose a waterbirth because they ‘*wanted the most natural birth possible*’ (P54 ^N,P^) which involved ‘*a natural experience*’(P250 ^N,P^) or a ‘*natural holistic approach*’ (P50 ^N,P^). One woman whose ‘*first birth was drug free on her back, wanted it to be even more natural*’ (P88 ^N,M^). Some women recognised ‘*The Birth Centre environment of low intervention midwifery-led care is more natural. Knew water good … calmer, softer way to birth baby*’ (P288 ^N,M^) or *‘I had a poor experience in private hospital with my other babies, I liked the model of care offered*’ (P210 ^Y,M^). Another woman wanted her main carer to be a ‘*midwife not obstetrician, I wanted natural environment, happy in public system of the Birth Centre environment, I wanted option to labour/birth in water*’ (P11 ^N,P^). One woman had experienced a previous traumatic birth and hoped water immersion might provide a more positive birth experience: ‘*First birth difficult – occiput posterior with trial of forceps. Had epidural at 8cm, wanted a simpler, more natural birth, a waterbirth was what I had hoped for*’ (P91 ^Y,M^). Another had ‘*failed to achieve a waterbirth with first birth, wanted natural birth*’ (P164 ^N,M^). Two women were midwives and one shared that ‘*I knew the background and research and had done waterbirth course. Work experience encouraged me and I knew there was less intervention with waterbirth*’ (P291^Y,P^). The other explained ‘*being a midwife I know waterbirth is gentle, I wanted the most natural birth possible*’ (P284 ^N,P^).

#### Relaxing environment

A total of 9% (27 of 296) of all women surveyed, 5% (5 of 93) of women who did and 11% (22 of 203) of women who did not birth in water, planned a waterbirth because they perceived it as promoting a relaxing birthing environment. The primary sentiments expressed by women in this theme included: ‘*it would be really relaxing and soothing for me*’ (P212 ^N,P^); *relaxation, I love water, feeling weightless*’ (P25 ^N,P^); it is a ‘*relaxing way to have baby, softening for body*’ (P287 ^N,P^); and ‘*I like baths, don't like idea of lying on back for birth, a peaceful, calm entrance into world for baby*’ (P26 ^Y,M^).

#### Recommended

A total of 8% (23 of 293) of all women surveyed, 14% (13 of 93) of women who did and 5% (10 of 203) of women who did not birth in water, opted for a waterbirth because it was recommended to them. The primary sentiments expressed by women in this theme included: ‘*My friend had a waterbirth and highly recommended it to me*’ (P216 ^Y,P^); ‘*I was influenced by sister who had two waterbirths at home, natural and beautiful*’ (P175 ^N,P^); ‘*Mum recommended waterbirth, had eight kids so lots of experience to share*’ (P195 ^Y,P^); ‘*Suggested by midwife, had only intended an active labour to begin with, mum is midwife, sister had two waterbirths*’ (P277^Y,M^); and ‘*I did a lot of reading up on waterbirth, I liked that wasn’t medical intervention, liked idea of being in warm bath*’ (P221^N,M^).

### Primary factor perceived by women to have contributed to their waterbirth

#### Women who achieved a waterbirth

**Fig. 2:**
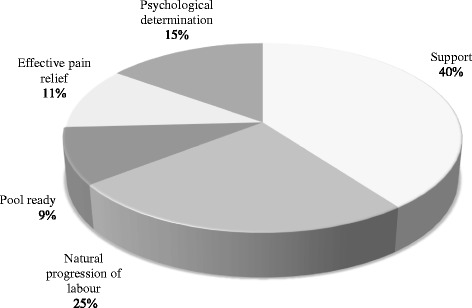
Primary factor perceived by women to have contributed to their waterbirth

Two fifths (40%; 37 of 93) of women suggested support as the primary reason they achieved a waterbirth, with the midwife named as the primary support person by 34 of 37 women who gave this response. A quarter (25%; 23 of 93) of women felt the fact their labour progressed without complication was the primary reason they achieved their desired waterbirth, whilst 15% (13 of 93) of women perceived their own psychological determination was the primary factor (Fig. 2).

#### Women who did not achieve a waterbirth

**Fig. 3:**
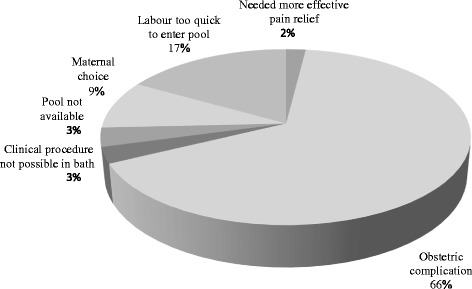
Primary factor perceived by women to have contributed to not achieving a waterbirth

Most (66%; 134 of 203) women who did not achieve a waterbirth perceived it was because they experienced an obstetric complication. The complications noted by women could occur in pregnancy such as a planned induction due to being post term, or a spontaneous labour at less than 37 weeks gestation. Alternatively, the complications noted by women could have occurred in labour, such as delayed progress, fetal distress, meconium stained liquor or fetal malposition. A further 17% (34 of 203) of women felt their labour progressed too quickly for them to enter the pool (Fig. 3).

#### Primary word women used to describe their birth

**Fig. 4:**
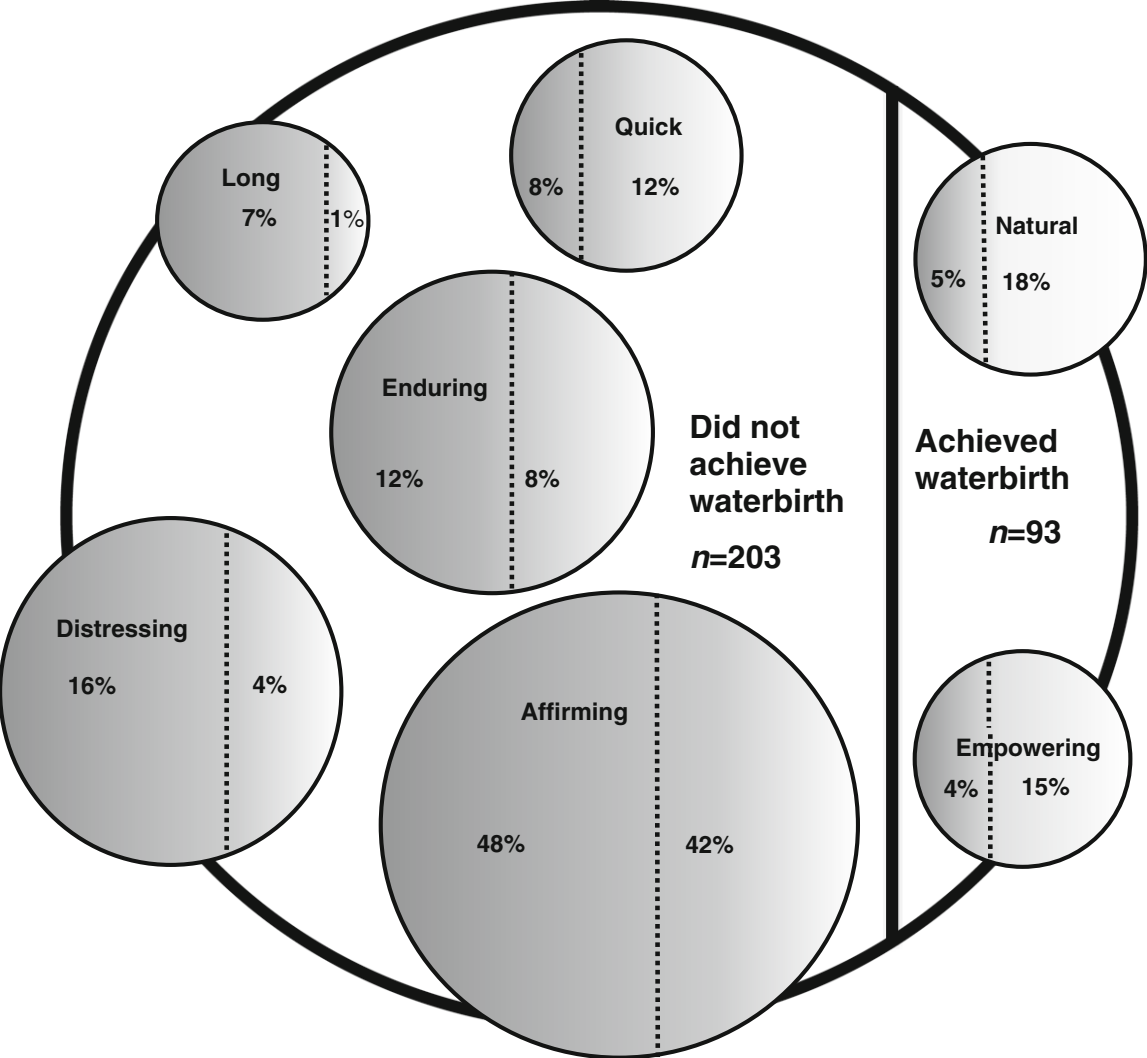
Primary word women used to describe their birth experience

Figure 4 describes the proportion of women who achieved or did not achieve a waterbirth in each of the coded themes (affirming, distressing, enduring, quick, natural, empowering, and long). A total of 46% (136 of 296) of all women surveyed, 42% (39 of 93) of women who did and 48% (97 of 203) of women who did not birth in water, used a word to describe their birth which was coded as affirming. These words included: positive; amazing; magical; easy; perfect; fantastic; great; awesome; surreal; wonderful; and beautiful.

A total of 12% (35 of 296) of all women surveyed, 4% (4 of 93) of women who did and 16% (32 of 203) of women who did not birth in water, used a primary word to describe their birth which was coded as distressing. These words included: disappointing; painful; intense; traumatic; scary; stressful; tortuous; devastating; and unexpected.

In addition, 11% (32 of 296) of all women surveyed, 8% (7 of 93) of women who did and 12% (24 of 203) of women who did not birth in water, used a primary word to describe their birth which was coded as enduring. These words included: enduring; intense; hard; tough; and challenging.

A total of 9% (26 of 296) of all women surveyed, 18% (17 of 93) of women who did and 5% (10 of 203) of women who did not birth in water, used a primary word to describe their labour which was coded as natural. These words included: calm; peaceful; gentle; relaxed; and intimate.

Similarly, 9% (26 of 296) of women, 12% (11 of 93) of women who did and 8% (16 of 203) of women who did not birth in water, used a primary word to describe their labour which was coded as quick. These words included: efficient; fast; and speedy.

A total of 8% (24 of 296) of all women surveyed, 15% (14 of 93) who did and 4% (8 of 203) who did not birth in water, used a primary word to describe their labour which was coded as empowering. These words included: empowering; control; and accomplished.

Finally, 5% (15 of 296) of all women surveyed, 1% (1 of 93) who did and 7% (14 of 203) who did not birth in water, proposed the primary word to describe their labour coded as long.

Analyses of the 45% (133 of 203) of women who did not achieve their planned waterbirth because they experienced an obstetric complication, found 44% (89 of 203) of these women used an affirming word to describe their birth, whilst 22% (45 of 203) used a distressing word.

## Discussion

An exploratory design using critical incident techniques enabled us to describe the perceptions and experiences of women who achieved or did not achieve their planned waterbirth. Overall, one third of women achieved a waterbirth and two thirds of women did not. It is clear not all women who set out to birth in water achieve their aim. To make an informed choice, women must be aware of the reasons why this birth preference may not occur. The primary reason women offered for planning a waterbirth was pain relief. Two fifths of women suggested support as the primary reason they achieved a waterbirth with the majority of women identifying the midwife as their primary support person. For women who did not achieve a waterbirth, two thirds perceived it was due to an obstetric complication. Our discussion will focus on how birth in water: incorporates a philosophy that labour is a normal healthy life event; enables the provision of individualised care which is responsive and respectful to changing needs; and meets a woman’s aspiration for a labour free of pharmacological analgesia.

There is limited understanding of what contributes to the health and wellbeing of women in labour [23], especially around care in water. Our exploration of what made women plan a birth in water found women wanted a natural birth in a relaxing environment where they could cope with their pain. The context of viewing labour as a healthy normal physiological event is dependent on a woman’s ability to focus on health not disease which aligns with salutogenesis. The theory of salutogenesis was originally described by a medical sociologist, Aaron Antonovsky, in the 1970s [24]. Antonovsky was interested in exploring what encourages individuals to maintain a healthy outlook [5]. Sense of coherence (SOC) is the foundation of salutogenesis [25]. An individual’s SOC represents the amount of self-esteem and confidence an individual exhibits [1], underpinning one’s ability to create health [26]. We surmise the 44% of women who did not achieve their planned waterbirth because they experienced an obstetric complication and used an affirming word to describe their birth had a high SOC. Although there is an absence of research linking like-minded women to midwives who support the salutogenic philosophy [1], what is clear is that salutogenesis is consistent with respectful midwifery care.

In our study setting waterbirth could only occur with the provision of low-risk midwifery-led care. It was difficult to separate these two symbiotic factors as they were intertwined. Therefore, an unsurprising finding was that two fifths of women perceived support was the primary reason they achieved a waterbirth, with the midwife named as the primary support person by the majority of these women. Respectful partnerships with women are a unique aspect of midwifery care as midwives provide women with information about what to expect, discuss expectations and involve them in decisions around their care [27]. Although respectful midwifery care is highlighted in *The Lancet’s* Midwifery series as a fundamental right of all women [8–10], it is challenging to provide waterbirth care without one to one support, as knowing the woman and her preferences for care during labour and birth are pivotal to waterbirth and building and sustaining respectful partnerships. A meta-analysis of continuous support for women in labour (including 22 RCTs with 15,288 women) found women who received continuous labour support were more likely to give birth spontaneously and less likely to use pain medications [28].

Most women experience some form of uterine discomfort during labour and concern about pain is common [29]. Women often enter labour with a preference for and against specific types of analgesia [30]. The buoyancy from immersion in water for labour and birth enables women to move freely, which has the propensity to relieve pain [1]. Therefore, it is not surprising that around half of the women we surveyed opted for a waterbirth as they perceived it would provide pain relief in labour. It has been suggested that the context of the birth environment can influence how women experience pain. When pain is perceived as productive and purposeful, it is more likely to be associated with positive cognitions [31]. Additionally, it has been found encouraging women to use pain relief methods where they are in control positively impacts the birth experience [30]. *The Lancet’s* Midwifery series highlights respectful evidence based care should include assessing a woman’s desire for non-pharmacological pain relief and encouraging her to adopt any upright position she finds comfortable in labour [10], two concepts synonymous with waterbirth. Indeed, a meta-analysis of maternal positions and mobility during first stage labour (including 25 RCTs with 5218 women) found women who remain upright or mobile in labour reported less pain [32].

### Limitations

The optimum time for recall around birth experience is unique to women and may have had an impact on our findings. The research methodology inhibited us from being able to assess the association between planning and achieving a waterbirth. For example, women who did not achieve a waterbirth were more likely than those who did to plan a waterbirth for pain relief. We could not associate this with an epidural. Similarly this methodology prevented us from exploring the specific characteristics women found supportive in their midwifery ‐led care and also the effect of parity. The sample was comprised of self-selected low risk women receiving care within a midwifery-led model of care in one birth centre who planned for water immersion during labour and birth, gave birth to a live infant and were willing to share perceptions of their experience. Although all women surveyed had birthed a live infant, we were unaware if they had birthed a healthy child; willingness to disclose this information during their interview was the woman’s choice. Therefore the context of the study must be considered when interpreting the transferability of the findings to other settings.

## Conclusion

This is the first study to explore the perceptions and experiences of women who achieved or did not achieve their planned waterbirth. Therefore, it initiates knowledge and understanding of the topic and is an important foci for future research, especially as it is clear not all women who set out to labour and birth in water achieve their aim and differences exist between the descriptions women provided about their experience. It is clear immersion in water for labour and birth facilitates a shift of focus from high risk obstetric-led care to low risk midwifery-led care. It also facilitates evidence based, respectful midwifery care which in turn optimises the potential for women to view their birthing experience through a positive lens.

## Abbreviations

IBM: International Business Machines
M: Multiparous
N: No
NY: New York
P: Primiparous
RCT: Randomised controlled trial
SOC: Sense of coherence
SPSS: Statistical Package for Social Sciences
WA: Western Australia
Y: Yes
